# Whipple’s Disease: A Diagnostic Challenge in Patients With Chronic Diarrhea

**DOI:** 10.7759/cureus.102988

**Published:** 2026-02-04

**Authors:** Elisa Veigas, João Lança Pereira, Marta Marques, Pedro Rodrigues, Jorge Correia

**Affiliations:** 1 Internal Medicine, Unidade Local de Saúde de Viseu Dão-Lafões, Viseu, PRT; 2 Infectious Diseases, Unidade Local de Saúde de Viseu Dão-Lafões, Viseu, PRT; 3 Anatomic Pathology, Unidade Local de Saúde de Viseu Dão-Lafões, Viseu, PRT

**Keywords:** chronic diarrhea, duodenal biopsy, tropheryma whipplei, weight loss, whipple's disease

## Abstract

Whipple’s disease, associated with chronic infection by *Tropheryma whipplei*, is an uncommon multisystem condition. It is frequently underdiagnosed because of its nonspecific and variable symptoms. Early recognition is important, as delays in diagnosis can result in multisystem involvement and potentially serious outcomes.

A 74-year-old man experienced chronic diarrhea, weight loss, and fatigue over three months. Initial investigations, including imaging and endoscopy, did not reveal any abnormalities. As symptoms persisted, a repeat endoscopy was performed after one month, showing multiple whitish elevated plaques in the duodenum. Histology identified periodic acid-Schiff and CD68-positive foamy macrophages with negative Ziehl-Neelsen staining, confirming Whipple’s disease. The patient was treated with ceftriaxone for two weeks, followed by a year of doxycycline and hydroxychloroquine, leading to rapid clinical remission. Due to ongoing histological changes, antibiotic therapy was continued for another year with trimethoprim-sulfamethoxazole and an additional six months of doxycycline. The patient remains asymptomatic on follow-up. Whipple’s disease has clinical features that overlap with other chronic gastrointestinal and rheumatologic disorders, often leading to diagnostic challenges. This case demonstrates the importance of maintaining a high index of suspicion and performing repeat endoscopic evaluation if symptoms continue despite unremarkable initial findings. Timely administration of suitable antibiotic therapy is associated with favorable clinical outcomes, even in cases with prolonged or atypical presentations.

## Introduction

Whipple’s disease (WD) is a rare condition, with an estimated incidence of 0.5-3 cases per million per year, and it is likely underdiagnosed due to its insidious and nonspecific clinical presentation [1,2]. Initial suspicion of the disease was reported in 1907 based on a gastric biopsy taken during autopsy, and for many years, diagnosis was made only after death. The first antemortem diagnosis occurred in 1947 [1,3].

The causative agent of WD is the Gram-positive bacterium *Tropheryma whipplei*, which is primarily found in sewage effluents and soil, suggesting a fecal-oral route of transmission. The condition mainly affects middle-aged men from rural areas in Europe, especially those exposed to contaminated environments through occupations such as agriculture or wastewater management [1-3].

The clinical spectrum is heterogeneous. Arthralgia is often the earliest manifestation and may precede gastrointestinal symptoms by years, frequently leading to diagnostic delay and misclassification as rheumatologic disease. Arthralgia is then followed by the onset of diarrhea, abdominal pain, and weight loss. WD may involve multiple organs, including the heart, lungs, eyes, and central nervous system, and can progress over several months to years [1,2]. Due to its broad range of clinical manifestations depending on the organ involved, it may resemble conditions such as seronegative rheumatoid arthritis (especially when arthritis is the sole presenting feature), sarcoidosis or lymphoma (if mediastinal lymphadenopathy is present), inflammatory bowel disease, vasculitis with intestinal involvement, or malabsorptive disorders like celiac disease [3-5]. Historically, untreated WD was uniformly fatal, whereas with appropriate antibiotic therapy, the mortality rate has dropped below 10% [1,5].

The pathophysiology of Whipple’s disease is not fully defined, but impaired host immunity appears to be central. Most individuals exposed to *T. whipplei* remain asymptomatic, whereas affected patients exhibit deficient macrophage bactericidal activity and an inadequate type 1 T cell response, allowing intracellular persistence of the organism. Genetic susceptibility has been suggested, including a higher frequency of HLA-B27 among patients. Accumulation of infected macrophages disrupts normal villous architecture, leading to malabsorption, and may facilitate systemic dissemination to other organs [6].

We describe a case of Whipple’s disease presenting exclusively with diarrhea and significant weight loss, without arthralgia or abdominal pain, in which the initial endoscopy was normal. This atypical and diagnostically challenging presentation underscores the importance of maintaining clinical suspicion and repeating endoscopic evaluation when symptoms persist. This study aimed to highlight these diagnostic pitfalls and the relevance of individualized treatment duration in cases with persistent histological abnormalities.

## Case presentation

A 74-year-old Portuguese man presented with chronic diarrhea, an 11-kg weight loss (17.5% of body weight), and asthenia over a three-month period. He reported two to three liquid bowel movements every other day, without blood, mucus, or dietary triggers. There was no fever, vomiting, or abdominal pain. He had previously worked in a gold factory in Germany and later as a construction contractor in Portugal, involving frequent exposure to outdoor environments. However, he had retired at the time of presentation. There was no history of recent travel, animal contact, or known exposure to wastewater sources. On physical examination, he appeared cachectic (body mass index of 19) and pale, with no evidence of lymphadenopathy or organomegaly. A comprehensive laboratory evaluation was undertaken, with the main findings detailed in Table 1.

**Table 1:** Laboratory results showing evolution from initial presentation to therapy and recovery. TSH: thyroid-stimulating hormone; ANA: antinuclear antibodies; ENA: extractable nuclear antigens; ASCA: anti-*Saccharomyces cerevisiae *antibodies; ESR: erythrocyte sedimentation rate; GDH: glutamate dehydrogenase; AFP: alpha-fetoprotein; CEA: carcinoembryonic antigen; PSA: prostate-specific antigen; CA 19-9: carbohydrate antigen 19-9

Laboratory parameters	6 months before	1 month of symptoms	3 months of symptoms	1 month of therapy	9 months of therapy	Reference range
Hemoglobin (g/dL)	12.3	11.2	9.2	12.1	15.5	12.5-17.2
Mean corpuscular volume (fL)	84	72	76	82	92	80-101
Iron (µg/dL)	-	16.9	8.1	34	67	50-150
Ferritin (ng/mL)	-	76	106	26	116	30-340
Transferrin (mg/dL)	-	157	118	233	180	180-380
Transferrin saturation (%)	-	7.4	4.5	11	27	20-45
Total protein (g/dL)	-	-	4.8	-	6.6	6.6-8.7
Albumin (g/dL)	-	-	2.8	-	4.1	3.5-5.0
Folic acid (ng/mL)	-	10	-	4.7	10	1.0-20.0
Vitamin B12 (pg/mL)	-	266	-	328	323	179-1130
TSH (mIU/L)	-	2.125	0.845	-	2.017	0.55-4.78
Free T3 (pg/mL)	-	2.9	-	-	-	2.0-4.2
Free T4 (ng/dL)	-	1.0	1.2	-	0.9	0.9-1.8
ANA/ENA screen	-	Negative	-	-	-	-
Anti-transglutaminase IgA; IgG (AU/mL)	-	-	Negative	-	-	-
Gastric parietal cell antibodies (U/mL)	-	-	<0.2	-	-	0-10
Intrinsic factor antibodies (U/mL)	-	-	<0.5	-	-	0-10
ASCA IgA; IgG (U/mL)	-	-	Negative	-	-	-
ESR (mm/h)	-	12	-	-	-	-
Fecal elastase (µg/g stool)	-	-	45	-	-	>200
Anti-endomysial IgA/IgG	-	-	Negative	-	-	-
Fecal calprotectin (mg/kg)	-	-	281	-	-	0-50
Stool culture (Shigella, Salmonella, Campylobacter)	-	-	Negative	-	-	-
*Clostridium difficile *GDH/toxins A and B	-	-	Negative	-	-	-
Stool parasitology	-	-	Negative	-	-	-
AFP (ng/mL)	-	-	<1.3	-	-	0-8.1
CEA (ng/mL)	-	-	3.0	-	-	0-5.0
CA 19-9 (U/mL)	-	-	6.1	-	-	0-37
Total PSA (ng/mL)	-	-	0.36	-	-	0-4

Laboratory findings included iron-deficiency anemia (hemoglobin: 9.2 g/dL; reference: 12.5-17.2 g/dL) with markedly reduced serum iron (8.1 µg/dL; reference: 50-150 µg/dL) and low transferrin saturation (4.5%; reference: 20-45%), together with hypoalbuminemia (2.8 g/dL; reference: 3.5-5.0 g/dL) (Table 1). Thyroid function was normal, and serologies for celiac disease and autoimmune gastritis were negative. Fecal calprotectin was mildly elevated, and fecal elastase was reduced (<200 µg/g), though both findings were inconclusive. Stool cultures and parasitological examinations were negative. Thoraco-abdominopelvic computed tomography, upper endoscopy, and colonoscopy revealed no abnormalities.

**Figure 1:**
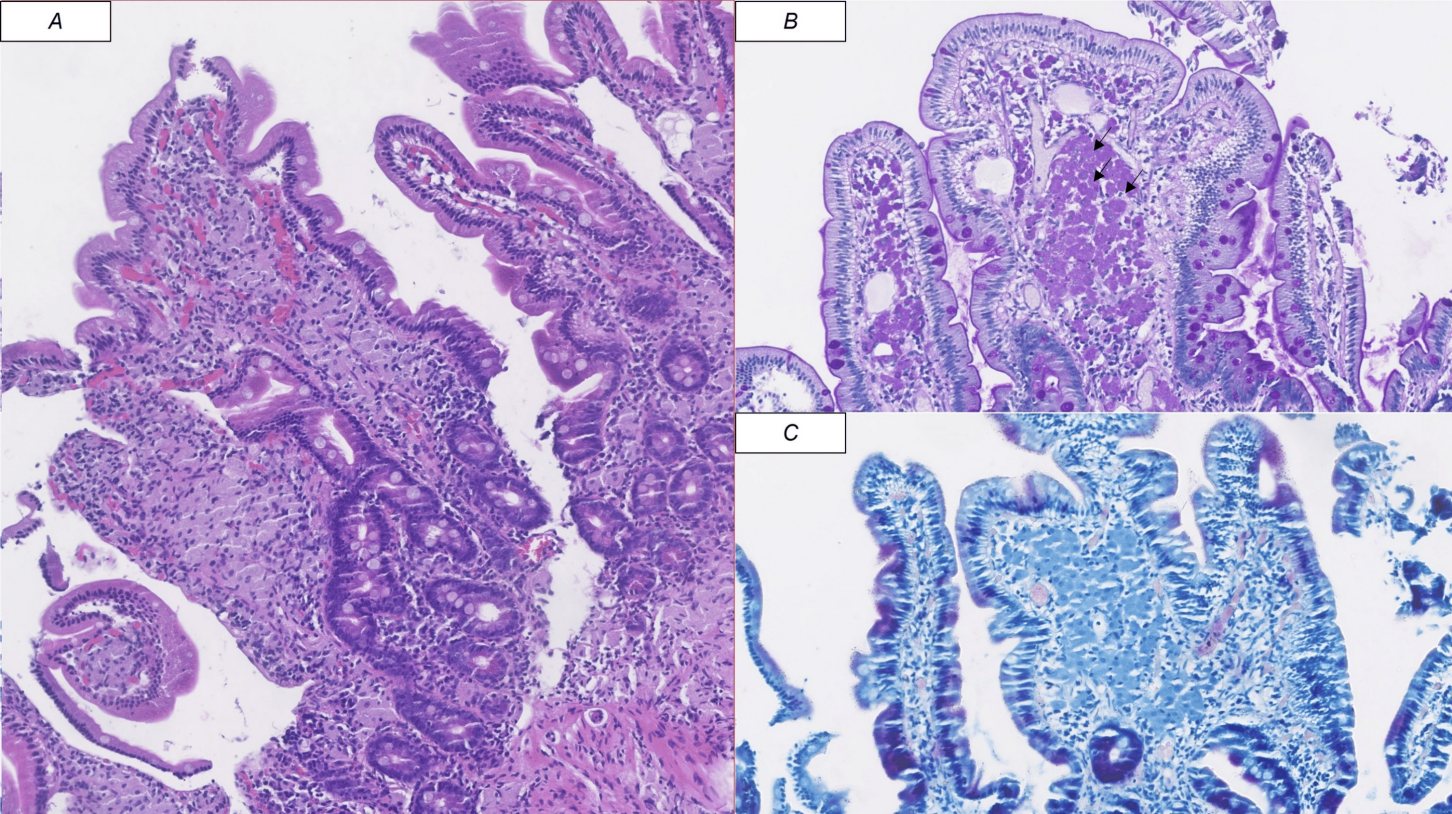
Immunohistochemical staining of duodenal biopsy specimens. Duodenal mucosal fragments with overall preserved villous architecture (A, hematoxylin and eosin stain). Occasional areas show expansion of the lamina propria by aggregates of epithelioid histiocytes with granular eosinophilic cytoplasm, positive for periodic acid-Schiff staining (B, arrows), and negative on Ziehl-Neelsen staining (C).

Because of persistent symptoms, a repeat upper endoscopy was performed one month later, which revealed multiple whitish elevated plaques in the duodenum. Histological analysis showed periodic acid-Schiff (PAS) and CD68-positive foamy macrophages and negative Ziehl-Neelsen staining, confirming the diagnosis of Whipple’s disease (Figures 1A-1C). This contrasted with the initial endoscopy, which had been completely unremarkable, underscoring how repeat evaluation may be essential when symptoms persist despite normal early findings.

Following this diagnosis, transthoracic echocardiography was performed to rule out cardiac involvement, showing no evidence of endocarditis. The patient received an induction regimen with intravenous ceftriaxone (2 g daily for two weeks), followed by oral doxycycline (200 mg/day) and hydroxychloroquine (600 mg/day) for one year, resulting in rapid clinical remission. Because histological abnormalities persisted at the first follow-up endoscopy, he was given a second induction course with intravenous meropenem (1 g every eight hours for 14 days), followed by another year of oral antibiotic therapy with trimethoprim-sulfamethoxazole (160/800 mg twice daily) and then an additional six months of doxycycline (200 mg/day).

At nine months of therapy (Table 1), hemoglobin, iron, and albumin had returned to within their normal reference ranges, indicating biochemical recovery consistent with the patient’s clinical improvement. He remained asymptomatic, with full weight recovery, normalization of laboratory parameters, and no recurrence of symptoms to date. Clinical, laboratory, imaging, and histological data were analyzed descriptively and interpreted in an integrated manner according to standard diagnostic criteria for Whipple’s disease.

## Discussion

Whipple’s disease may evolve over months to years, with a median of seven years from initial symptoms [5,7]. Arthralgia is often the first manifestation and may be misleading, as nearly half of patients are initially treated for presumed rheumatologic disease before the correct diagnosis of Whipple’s disease is established [7]. Our patient presented exclusively with digestive complaints and laboratory evidence of malabsorption. This atypical presentation highlights the variability of disease expression. The patient’s age and male sex are consistent with the typical demographic described for Whipple’s disease, although his background in construction rather than agriculture reflects how environmental exposure may be indirect or incidental.

Diagnosis relies on clinical suspicion confirmed by histopathological or molecular findings. In most cases, tissue is obtained from duodenal biopsies, but other specimens, such as synovial fluid, cerebrospinal fluid, or cardiac valve tissue, may be used depending on organ involvement. In small bowel disease, upper endoscopy typically shows a pale yellow or whitish mucosa with thickened folds and whitish confluent elevated plaques alternating with friable areas [1,3]. Small bowel biopsies demonstrate expansion of the lamina propria by aggregates of foamy CD68-positive macrophages containing PAS-positive bacilli inclusions. The absence of acid-fast organisms on Ziehl-Neelsen staining excludes *Mycobacterium avium* and *M. tuberculosis* infections [1,2]. However, biopsies may appear normal in the early stages of the disease [3]. In our case, the initial endoscopy and biopsies were unremarkable, and only repeat evaluation revealed characteristic lesions.

The persistence of PAS-positive macrophages after therapy does not always indicate ongoing infection, as residual bacterial material may remain for years despite microbiological cure. Additionally, asymptomatic colonization of *T. whipplei* in the gastrointestinal or respiratory tract can lead to isolated PCR positivity in the absence of clinical disease [5]. *T. whipplei *may colonize the gastrointestinal tract of healthy individuals, with bacterial DNA detected in 6.9% of fecal samples and 15.2% of saliva samples [8]. As a result, treatment decisions should be guided by clinical progression, histological findings, and microbiological results [5]. In our patient, the persistence of PAS-positive macrophages after two years of adequate therapy mirrors these findings. Given the absence of symptoms and normalization of laboratory parameters, these persistent histological abnormalities may represent histologic scarring or residual bacterial components rather than active infection (absence of symptoms or systemic inflammation).

In this patient, the initial upper endoscopy was unremarkable, with characteristic lesions identified only on repeat examination one month later. PCR confirmation of *T. whipplei *was not performed due to unavailability at the time of diagnosis, which may represent a limitation. Histological abnormalities persisted at both one- and two-year follow-up periods, despite complete clinical remission. As a precautionary measure, antibiotic therapy was continued for a total of two years, including a final six-month course of doxycycline, with close clinical monitoring. The prognostic significance of persistent histological abnormalities following adequate therapy remains unclear, as no definitive correlation has been established. The decision to prolong therapy was made in the context of uncertain evidence regarding the prognostic meaning of isolated histological persistence. Available recommendations support long-term regimens with agents that achieve adequate tissue and CNS penetration, but they do not specify a uniform duration of therapy when histological abnormalities persist despite full clinical remission [1,5]. Our approach, therefore, remained consistent with published treatment principles while relying on clinical judgment in an area where evidence is limited.

Although potentially fatal if untreated, the prognosis is excellent once appropriate antibiotics are initiated, with rapid clinical remission and improved quality of life within weeks [1-3]. Clinical relapse occurs in up to 30% of patients [5]. Optimal therapy is not firmly established due to the limited number of cases. Current regimens emphasize antibiotics with good central nervous system penetration. A commonly used protocol includes induction with intravenous ceftriaxone 2 g daily for two weeks (or meropenem 1 g three times daily), followed by oral trimethoprim-sulfamethoxazole 160/800 mg twice daily for at least one year, or alternatively, hydroxychloroquine 600 mg/day plus doxycycline 200 mg/day [1-4,9]. Recent evidence suggests that an entirely oral regimen with trimethoprim-sulfamethoxazole may be non-inferior to those including an intravenous induction phase [4]. Lifelong doxycycline 200 mg/day may be considered in patients with recurrent disease [1-4]. In the present case, therapy followed these recommendations, with an initial intravenous induction phase followed by prolonged oral regimens. The decision to extend treatment beyond two years, despite clinical remission, was based on persistent histologic abnormalities and the absence of clear prognostic guidance regarding their significance.

## Conclusions

Whipple’s disease remains a diagnostic challenge due to its protean manifestations and its ability to mimic more common conditions. Prompt recognition and appropriate antibiotic therapy are crucial for favorable outcomes. In our case, the persistence of histological abnormalities after clinical remission prompted prolonged treatment, which was well tolerated and associated with sustained recovery. Lifelong clinical monitoring is advisable given the risk of relapse and the uncertain significance of persistent PAS-positive macrophages.

This case reinforces the need for further studies to clarify the prognostic significance of persistent histological abnormalities and to define optimal treatment duration. It also demonstrates that repeat endoscopy may be crucial in patients with persistent, unexplained symptoms despite an initially normal evaluation. These findings emphasize the importance of individualized diagnostic and therapeutic strategies in Whipple’s disease.
